# Phosphatidylethanolamine N-methyltransferase: from Functions to Diseases

**DOI:** 10.14336/AD.2022.1025

**Published:** 2023-06-01

**Authors:** Jiayu Li, Yanguo Xin, Jingye Li, Hui Chen, Hongwei Li

**Affiliations:** ^1^Department of Cardiology, Cardiovascular Center, Beijing Friendship Hospital, Capital Medical University, Beijing, China.; ^2^Beijing Key Laboratory of Metabolic Disorder Related Cardiovascular Disease, Beijing, China.

**Keywords:** phosphatidylethanolamine, phosphatidylcholine, PEMT, obesity, choline

## Abstract

Locating on endoplasmic reticulum and mitochondria associated membrane, Phosphatidylethanolamine N-methyltransferase (PEMT), catalyzes phosphatidylethanolamine methylation to phosphatidylcholine. As the only endogenous pathway for choline biosynthesis in mammals, the dysregulation of PEMT can lead to imbalance of phospholipid metabolism. Dysregulation of phospholipid metabolism in the liver or heart can lead to deposition of toxic lipid species that adversely result in dysfunction of hepatocyte/cardiomyocyte. Studies have shown that PEMT^-/-^ mice increased susceptibility of diet-induced fatty liver and steatohepatitis. However, knockout of PEMT protects against diet-induced atherosclerosis, diet-induced obesity, and insulin resistance. Thus, novel insights to the function of PEMT in various organs should be summarized. Here, we reviewed the structural and functional properties of PEMT, highlighting its role in the pathogenesis of obesity, liver diseases, cardiovascular diseases, and other conditions.

## Introduction

**Figure 1.**
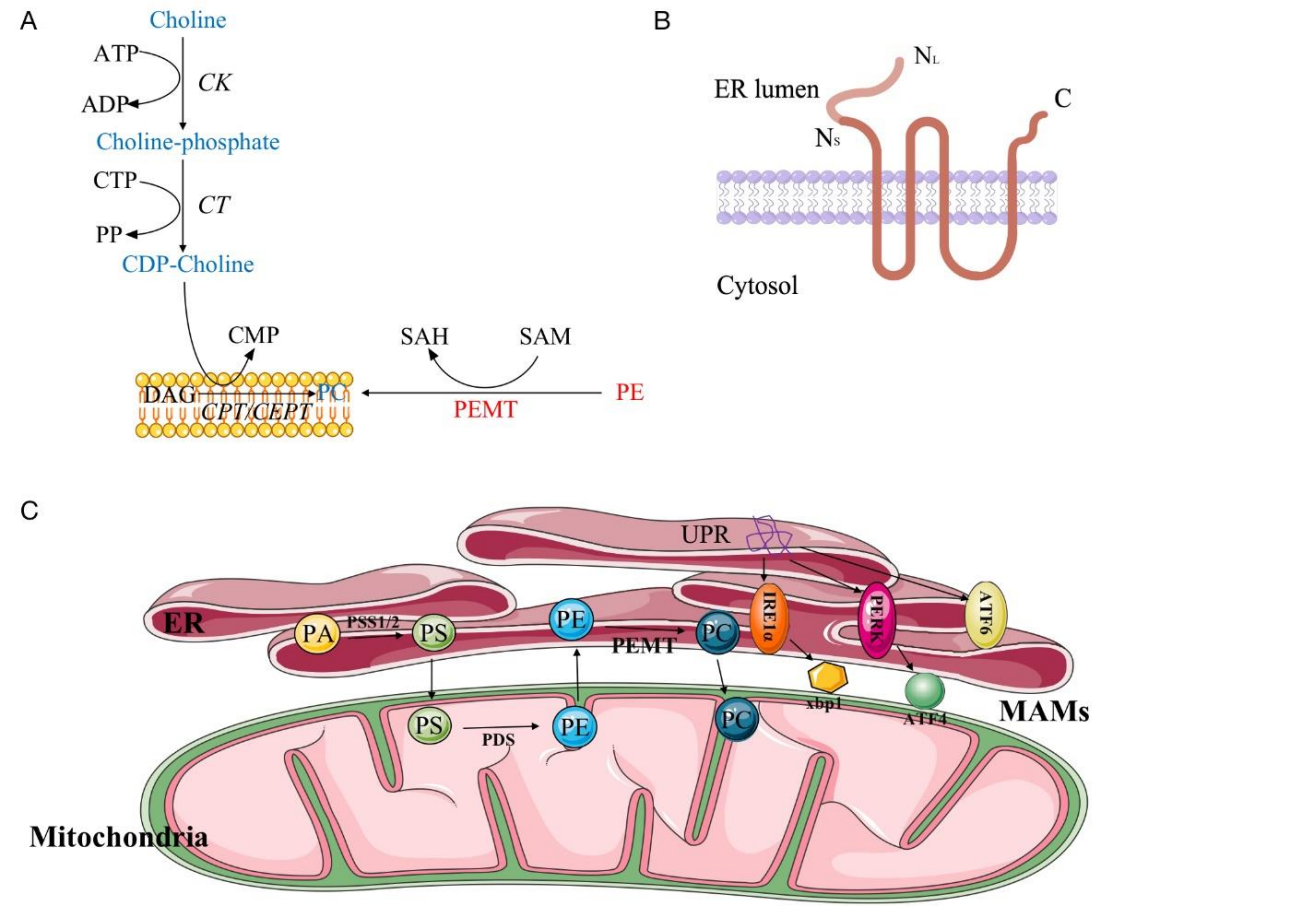
Structural and functional roles of PEMT. (A) CDP-choline and PEMT pathways for synthesis of phosphatidylcholine. (Enzymes indentified are CK, choline kinase; CT, CTP-choline phosphate cytidylyltransferase; CPT/CEPT, CDP-choline phosphotransferase.) (B) Topological model of PEMT in the ER membrane. (N refers to amino terminus and C to the carboxyl terminus of PEMT. Variant 1 encodes PEMT-L with N_L_ terminus and variant 2, 3 encode PEMT-S with N_S_ terminus.) (C) The role of PEMT between ER and MAMs. (UPR is started to clear the ER of misfolded or unfolded proteins and restore ER homeostasis under various conditions causing ER stress, with the c-ATF6, PERK-ATF4 and IRE-Xbp1s signal pathways. PA in ER is catalyzed into PS by PSS1 and PSS2. Then the PS directly moves to mitochondria via the physical contact for its carboxylation to PE. PE in mitochondria is transferred back to ER and methylated into PC via PEMT.)

Phospholipids are a class of lipids that consist of two fatty acids, a phosphate group, and a glycerol molecule. Phospholipid metabolism plays a crucial role in cellular physiology. Moreover, phospholipids are a key component of cell membranes, forming lipid bilayers, and play vital roles in cellular functional processes. Phosphatidylcholine (PC) is an important phospholipid [1] that incorporates choline as a headgroup and comprises nearly 40-50% of cellular phospholipids. In addition to forming cell membranes, PC is also necessary for the biosynthesis of lipoproteins, lung alveolar surfactants, and bile. In mammals, two major pathways for PC biosynthesis have been discovered. In the 1950s, Eugene Kennedy and colleagues reported the CDP-choline pathway in PC synthesis[2, 3]; all the steps of the pathway occur in the nucleus, and CTP: phosphocholine cytidylyltransferase is the rate-limiting enzyme. Choline is an essential dietary amine for the human body and serves as a source of methyl groups for various metabolic steps [4]. In addition, choline plays a vital role in the regulation of gene expression, membrane signaling, and lipid metabolism. Food is an important source of choline [5], but humans can also produce endogenous choline through a de novo synthesis pathway; the CDP-choline pathway contributes to PC synthesis even in the absence of exogenous choline. An additional pathway, mainly occurring in the liver, catalyzes three sequential methylation reactions by converting phosph-atidylethanolamine (PE) to PC [6], which is the only endogenous pathway for choline biosynthesis in mammals (Fig. 1A). The key enzyme involved in this process is phosphatidylethanolamine N-methyltransferase (PEMT). Approximately 30% of the PC is generated via the PEMT reaction in the liver, and the remaining PC is generated via the CDP-choline pathway in the liver [1, 7]. Despite contributing to a high proportion of total PC, the specific function of PEMT-mediated PC remains unclear. Walkey et al. established PEMT knockout mice (PEMT^-/-^) and found that they display no abnormal phenotype and PEMT knockout does not affect the serum lipid levels, including PE and PC [8, 9]. However, PEMT^-/-^ mice cannot survive on a choline-deficient diet [10], indicating that PC from the PEMT pathway is indispensable for pathological processes. A previous study reported that PEMT only exists in the liver, but the recent study proved that PEMT is also expressed in the brain, testis, heart, and skeletal muscle [11]. However, the underlying role of PEMT in these organs remains unclear. Further studies exploring the physiological functions of PEMT and the relationship between PEMT and pathological processes are necessary. Here, we briefly discuss the structural and functional roles of PEMT. In particular, we have summarized the relationship between PEMT and pathological conditions.

### PEMT: structural insights

There are two isoforms of PEMT: PEMT1 and PEMT2. PEMT1 is associated with endoplasmic reticulum (ER) [12], and PEMT2 is localized in the mitochondria-associated membrane [13]. PEMT1 and PEMT2 are encoded by the same gene but have different posttranslational modifications or alternative splicing; the possibility of differential gene splicing and mRNA editing cannot be eliminated as well. The human PEMT gene is located on chromosome 17p11.2 and contains nine exons, eight introns, and different promoters that generate three transcripts [11]. Current evidence indicates that alternative splicing of PEMT exons 1, 2, and 3 is human-specific [14]. Exon 2-containing transcripts are the major forms that exist in the liver. Variant 1 encodes long isoform PEMT (PEMT-L) and variants 2 and 3 encode the short isoform PEMT (PEMT-S). All three transcripts are found in the liver, but only exon 1-containing transcript is present in the heart[11]. Both PEMT-L and PEMT-S are located in the ER, and the activity of PEMT-S is much higher than that of PEMT-L (Fig. 1B). Although PEMT-L and PEMT-S significantly increases the production of PC, overexpression of PEMT-S can lower the level of PE; however, overexpression of PEMT-L has no impact on this process [15]. Shields et al.[16] reported that PEMT consists of four transmembrane regions on the ER membrane, with the N- and C-termini located in the cytosol (Fig. 1B).

### PEMT and mitochondria

Mitochondria are double-membrane-bound organelles, and PE and PC are the two main types of phospholipids in the mitochondrial membrane, although the percentage of their composition may be slightly different in different organs[17, 18]. The distribution of PE and PC is distinctive. The mitochondrial matrix is separated from the cytosol by two membranous structures: the outer mitochondrial membrane (OMM) and inner mitochondrial membrane (IMM). Approximately 70% of the PE is located on the outer side of the OMM, whereas PC is uniformly located on both sides of the OMM [19]. Mitochondria are semiautonomous organelles that can synthesize PE and PC by themselves. Cardiolipin is the principal glycerophospholipid of the mitochondrial membrane, which consists of a double glycerophosphate backbone and four fatty acyl side chains [20]. As a mitochondria-exclusive phospholipid, cardiolipin has been reported to participate in various pathogenic processes by interacting with the electron transport chain and mitochondrial dynamics [21]. Current evidence indicates that PE and cardiolipin deficiency may inhibit the mitochondrial fusion process and interrupt mitochondrial physical function. More importantly, mitochondria are also the main vendors of PE for other organelles [22]; the deficiency of PE cannot be compensated by other metabolic pathways outside the mitochondria [23, 24]. Steenbergen et al. reported that elimination of PE production in mitochondria via phosphatidylserine (PS) decarboxylase silencing may cause embryonic mortality between days 8 and 10 of embryonic development [25], and the CDP-choline pathway cannot substitute for the lack of PS decarboxylase. Veen et al. [26] discovered that mitochondria are smaller and more elongated in PEMT^-/-^ mice. Mitochondrial respiration activity increases in hepatocytes lacking PEMT, which is characterized by increasing activities of cytochrome c oxidase and succinate reductase. In addition, mitochondrial PE depletion in Chinese hamster ovary cells was found to change mitochondrial morphology and decrease respiratory capacity [27]. The PEMT pathway can maintain the intracellular level of liver receptor homolog-1 (LRH-1), which is a nuclear receptor that binds to the promoter and regulatory regions of target genes [28]. The PEMT pathway is closely dependent on S-adenosylmethionine (SAM) metabolism, and the ratio of SAM to S-adenosylhomocysteine provides a key index of the endogenous methyl pool. LRH-1 acts as a sensor for the state of SAM metabolism and a regulator of the methyl pool[29]. The PEMT-LRH-1 pathway regulates mitochondrial biogenesis in hepatocytes [30]. SAM is required for the PEMT pathway, and Choi et al. [29] found that SAM supplementation increases LRH-1 activation in a PEMT-dependent manner. In summary, these studies demonstrated that even a moderate reduction in mitochondrial PE can substantially alter mitochondrial functions.

### PEMT and ER

ER is an important eukaryotic organelle that contains a network of flattened tubules. It plays a vital role in lipid production, processes, and transportation [31]. Around 60% of PCs is generated from the ER, a crucial member of the eukaryotic cellular membrane [32]. Therefore, internal homeostasis is a prerequisite for ER function. Among the various conditions that cause ER stress [33], impaired protein folding in the ER leads to an accumulation of misfolded proteins, which induces the unfolded protein response (UPR). The UPR is a conserved program that clears the misfolded or unfolded proteins in the ER and restores ER homeostasis [34]. There are three major UPR pathways initiated in the ER [35]: the c-ATF6, PERK-ATF4, and IRE-Xbp1s signaling pathways (Fig. 1C).

PC is essential for maintaining physical ER function [32]. The PC/PE ratio may change the cellular integrity and lead to rapid liver failure in PEMT^-/-^ mice [36]. Gao et al.[37] measured the hepatic PC/PE ratio in the ER of chow-fed PEMT^-/-^ mice, which was found to be lower than that of wild type mice. In addition, a lower PC/PE ratio results in hepatic ER stress. However, upregulation of PEMT also initiates hepatic ER stress via a distinct pathway[38]. An increased PC/PE ratio impairs ER homeostasis via endogenous sarco/endoplasmic reticulum Ca^2+^-ATPase (SERCA) dysfunction. Correcting the ER PC/PE ratio by suppressing liver PEMT expression can significantly relieve ER stress. Downregulation of PEMT has also been reported to ameliorate ER stress and reverse apoptosis in diabetic nephropathy [39]. Free choline is typically utilized to produce PC via the Kennedy pathway and blocking PC production can induce ER stress. However, Kimata et al.[40] found that another methyltransferase, Opi3, is also important in maintaining the production of PC. Opi3 mutation induces an UPR. We believe that PEMT plays a vital role in maintaining the PC/PE ratio. Both upregulation and downregulation can disturb intracellular homeostasis.

### PEMT and MAMs

The physical contact between highly dynamic and regulated membranes forming a specific closed structure between intracellular organelles is indispensable for maintaining their physical functions [41]. The ER and mitochondria are juxtaposed to form mitochondria-associated membranes (MAMs). In the 1950s, Biophys et al. first reported MAMs as a physical domain between the ER and mitochondria [42]. Decades later, Vance isolated a membrane fraction associated with mitochondria, which strengthened the study on MAMs [43]. Transmission electron microscopy enabled the observation of the detailed structure of MAMs; the average distance between the ER and mitochondria was found to vary from 10-60 nm [44]. Increasing evidence have indicated that MAMs are associated with the regulation of numerous physical functions, including calcium signaling, autophagy, regulation of phospholipid synthesis by mitochondrial dynamics, inflammation, and cell death [45-47].

MAMs are enriched with lipid-related enzymes, such as PS synthase 1 and 2 (PSS1/2)[48]. PEMT2 is also a specific marker protein for MAMs. MAMs act as dynamic platforms for phospholipid synthesis. Phosphatidic acid (PA) in the ER is catalyzed into PS by PSS1 and PSS2. Then, PS directly moves to the mitochondria through physical contact for its carboxylation to PE. PE in the mitochondria is transferred back to the ER and methylated into PC by PEMT2 (Fig. 1C) [49, 50]. A previous study has indicated that defective MAMs may result in PC defects and abnormal lipid accumulation [51]. In addition, PC synthesis in MAMs involves many factors such as ATP, calcium, magnesium, and enzymes, which can regulate physical activity [52, 53].

### PEMT and diseases

**Figure 2.**
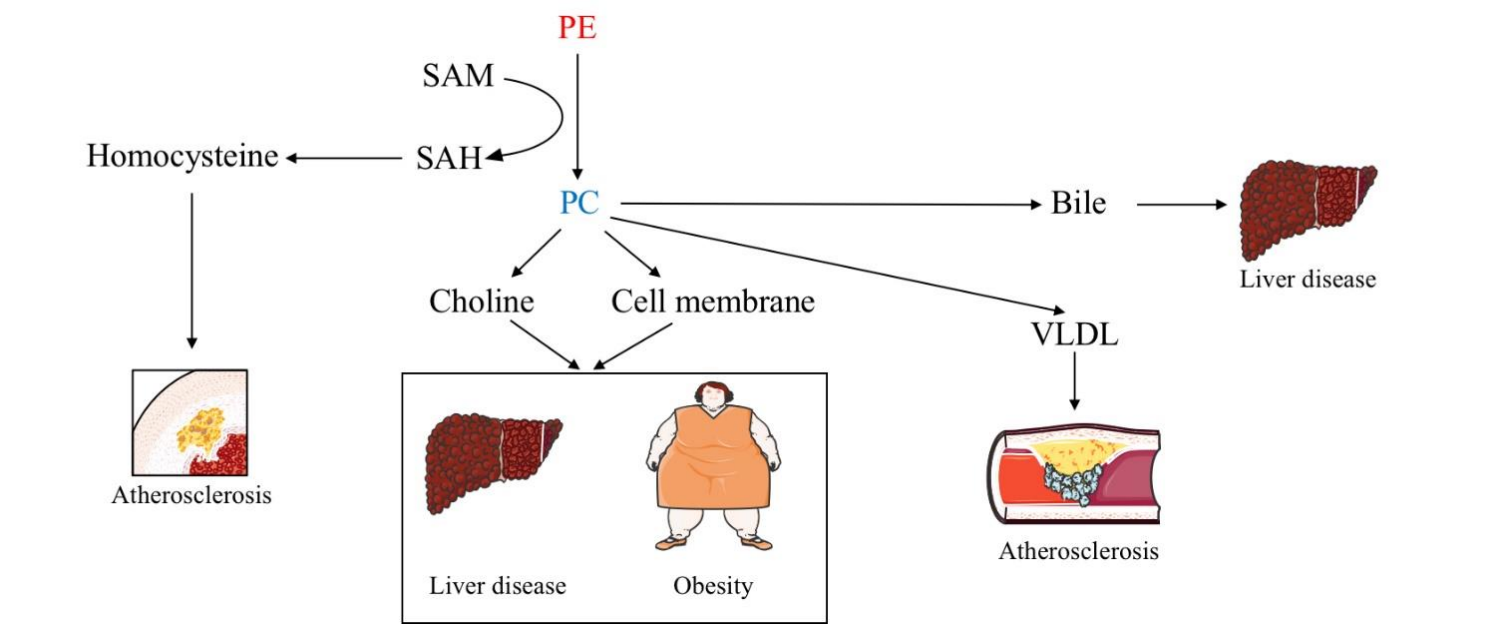
Potential mechanisms of PEMT in obesity, non-alcoholic liver disease and cardiovascular diseases. As the source of PC, PE metabolism relys largely on SAM, the production of SAH, homocysteine contributed to the process of atherosclerosis; high level of PC contributed to various pathological processes, such as liver diseases, obesity, and accumulation of VLDL, followed by atherosclerosis.

The importance of lipid metabolism in physiology confirms the impact of PEMT on the development of different pathological conditions. In this paper, we summarize the main findings that have reported a link between PEMT alterations and several diseases (Fig. 2) (Table 1).

### PEMT, Obesity, and non-alcoholic fatty liver diseases

Obesity is a global public health problem, with over 500 million people suffering from obesity worldwide [54], leading to the development of metabolic syndrome and related comorbidities such as type 2 diabetes mellitus (T2DM), non-alcoholic fatty liver disease (NAFLD), and cardiovascular diseases [55]. In a study that included 372 patients, Chen et al. found that diabetic patients with PEMT G774C polymorphism suffer a higher risk of microangiopathy [56] (Table 1). In addition, PEMT deficiency can suppress ER stress and ameliorate diabetic nephropathy [39] (Table 1). NAFLD is an important manifestation of metabolic syndrome and the most common cause of abnormal liver function, which can progress to hepatocyte necrosis, liver fibrosis, and cirrhosis [57]. The mechanisms underlying NAFLD are not well understood. Excess triglyceride (TG) accumulation in the liver accounts for steatosis; therefore, PC deficiency may disturb the hepatic secretion of triacylglycerols [58-60], indicating that PC metabolism is associated with NAFLD [61, 62]. As mentioned previously, approximately 30% of the PC in the liver is produced via the PEMT pathway. Wan et al. found that PEMT^-/-^ mice can survive from high-fat diet (HFD)-induced obesity but exhibit severe non-alcoholic steatohepatitis with a decreased PC/PE ratio [63] (Table 1). PEMT is the key one-carbon cycle protein, converting PE to PC with the help of hepatic SAM as a methyl donor. Lower liver SAM or PEMT^-/-^ decreases PC production, followed by impaired very low-density lipoprotein (VLDL) secretion, which causes high TG accumulation in hepatocytes [64-66]. Moreover, Nakatsuka et al. [67] found that PEMT can bind with the clathrin heavy chains and P53 anti-oncogene, inhibiting the transcription of P53, which may induce the development of liver tumors. PC synthesized in hepatocytes is secreted into bile, and the daily secretion of PC is almost equal to the hepatic PC content[68]. The multidrug resistance 2 (Mdr2) gene, which encodes a canalicular membrane P-glycoprotein, is essential for hepatic PC secretion. It acts by translocating PC from the inner to the outer leaflet of the canalicular membrane of hepatocytes [69, 70]. Similar to PEMT^-/-^mice, Mdr2^-/-^ mice showed intrahepatic cholestasis via the disruption of PC secretion into bile [68]. However, Mdr2^-/-^/PEMT^-/-^ mice escape liver failure by maintaining a normal PC/PE ratio [71]. In PC12 cells, choline deprivation causes a decrease in PC, but not PE, leading to cell death [72]. Additionally, an increase in PE, but not PC, may damage cardiomyocytes by leakage of lactate dehydrogenase (LDH) [73]. Thus, the PC/PE ratio is a critical modulator of membrane integrity. A decrease in the PC/PE ratio is correlated with a decrease in the membrane potential of hepatocytes [71], which may initiate inflammation due to the flow of ions and cytokines[74, 75]. Presa et al. reported that PEMT^-/-^ mice fed with vitamin E, an antioxidant, showed improved VLDL-TG secretion but not reduced hepatic TG content by restoring acid ceramidase (Asah1) and ceramide kinase (Cerk) levels [76]. Veen et al. [77] showed that hepatic inflammation in PEMT^-/-^ mice treated with pioglitazone can be attenuated via peroxisomal proliferator-activated receptor-γ (PPARγ). In addition, Gao et al. reported that the hepatic vagus nerve may be involved in steatohepatitis in PEMT^-/-^ mice fed with HFD; hepatic vagotomy normalizes phospholipid content and attenuates steatohepatitis by increasing the anti-inflammatory cytokine interleukin-10 (IL-10)[78].

**Table 1:** Roles of PEMT in diseases.

	Disease	Correlations/Functions	Potential mechanisms
Metabolic Diseases	T2DM	ameliorate diabetic nephropathy with deficiency of PEMT	suppress ER stress [39]
	T2DM	higher risk of microangiopathy [56]	-
	NAFLD	survive from high-fat diet-induced obesity with deficiency of PEMT	disturb secretion of triacylglycerols [63]
	central obesity	rs936108-C in the PEMT locus is associated with waist circumference and waist-to-hip ratio [94]	-
Cardiovascular Diseases	CAD	related to RAI1-PEMT-RASD1 in the PEMT locus [95]	-
	HTx	temporal relationship between PEMT gene expression in the human heart and clinical characteristics of heart transplantation (HTx) [97]	-
	AS/lipotoxic cardiomyopathy	decrease the risk of atherosclerosis and lipotoxic cardiomyopathy with deficiency of PEMT	eliminate PC biosynthesis, reduce TG and cholesterol levels [100]
	Hyperhomocysteinemia	decrease circulating Hcy by approximately 50% with deficiency of PEMT	reduce the product of S-adenosylhomocysteine [107, 108]
Nervous System Diseases	AD	contribute to cerebrovascular and neurodegenerative changes	AdoHcy increases with age, inhibits hepatic PEMT activity, decrease flux of docosahexaenoic acid [119]
	neural development	effect fetal brain development with deficiency of PEMT	increase progenitor cell mitosis and protein methylation, decrease calretinin levels [122, 126]
	neural development	effect fetal brain development with deficiency of PEMT	change of docosahexaenoic acid-containing phospholipids [123]
	NTD	linked with SNP, PEMT rs7946 [127]	-
Cancer	liver tumors	inhibit the transcription of P53, induce the development of liver tumors	bind with the clathrin heavy chains and P53 anti-oncogene [68]
	liver tumors	tumor suppressor	overexpression of PEMT2 partially reverses the growth of hepatoma cells [130]

PEMT: Phosphatidylethanolamine N-methyltransferase; T2DM: type 2 diabetes mellitus; ER: endoplasmic reticulum; NAFLD: nonalcoholic fatty liver disease; CAD: coronary artery disease; HTx: heart transplantation; AS: atherosclerosis; PC: Phosphatidylcholine; TG: triglycerides; AD: Alzheimer’s disease; AdoHcy: S-adenosylhomocysteine; NTD: neural tube defect.

Premenopausal women receiving a low-choline diet are less likely to develop steatohepatitis than men and postmenopausal women, and choline deprivation may increase PEMT activity in the liver of male rats but not female rats[79-81]. These results indicate that estrogen levels may contribute to PEMT activity. Moreover, estrogen can increase PEMT transcription in hepatocytes after 17-β-estradiol treatment[82]. In addition, the PEMT gene contains estrogen response elements (EREs) in its promoter regions. Estrogen interacts with its receptors, ERα and ERβ[83], which bind to EREs to regulate the expression of PEMT. This may explain why premenopausal females fed with a low-choline diet may not suffer organ dysfunction; hepatic PEMT can catalyze the de novo biosynthesis of choline. Not all genes are regulated by estrogen through interactions with EREs. Estrogen can also regulate target genes by interacting with transcription factors[84]. Using the 3T3-L1 adipocytes model, Laura et al. found that transcription factors Sp1, Sp3, and YY1 can regulate the expression of PEMT by binding to its regulatory region[85].

VLDL can transfer TG from the liver to peripheral plasma, whereas other types of lipoproteins, such as high-density lipoproteins (HDL) can remove cholesterol from the plasma to the liver, which is believed to be “good” cholesterol. PEMT^-/-^ mice demonstrated an increased uptake of HDL in the liver. To explore the underlying mechanisms, Robichaud et al.[86] measured the amount of hepatic scavenger receptor class B type 1 (SR-BI) and found a significant increase in SR-BI. SR-BI is a specific HDL receptor located on the surface of hepatocytes that selectively uptakes lipids from plasma [87, 88]. They found that PEMT^-/-^ mice showed an increased expression of hepatic PDZK1, an important SR-BI regulator that interacts with the C-terminal cytoplasmic domain of SR-BI. Increased SR-BI accounts for the high uptake of HDL.

### PEMT and cardiovascular diseases

Panagia et al. [89] confirmed the presence of PE N-methylation activity, which catalyzes PE to PC, in the sarcolemma, mitochondria, and microsomes of rat heart. Thereafter, the relationship between PEMT and the heart has been increasingly investigated. Taira et al. [90] observed PEMT activities in rat heart and found that the total methylation activity for catalytic site I in sarcolemma and sarcoplasmic reticulum is stimulated within 2 min by isoproterenol, an agonist for cardiomyocyte hypertrophy, in a dose-dependent manner. Similarly, the PEMT activity in rat heart first increases and then decreases after an intraperitoneal injection of isoproterenol [90]. Pre-treatment with atenolol, a beta-blocking drug, prevents isoproterenol-stimulated methylation levels and isoproterenol-induced changes in hemodynamic parameters. Beta-adrenoceptor antagonists interact with the sarcolemma and sarcoplasmic reticular membranes to exert negative inotropic action by affecting Ca^2+^ fluxes in myocardial cells. Panagia et al. [91, 92] observed that propranolol, acebutolol, and other beta-blocking drugs inhibit the methylation level of PEMT at low concentrations. Both propranolol and acebutolol change the methyltransferase activities at site II in rat heart sarcolemma, but not in the mitochondria. The effect of beta-adrenoceptor antagonists on PEMT activity is probably linked to the mode of action of the drugs. The standpoint is supported by Tappia’s findings[93], that is, Ca^2+^-pump inhibitors, including verapamil and diltiazem, can suppress PE N-methylation. Inhibition of PE N-methylation at site III may serve as a biochemical mechanism for the inhibition of cardiac Ca^2+^ pumps and altered cardiac function. This indicates a connection between PEMT and cardiovascular diseases. Although each PEMT transcript is present in a different amount in the liver, the heart and testis contain only one or two transcripts, respectively [14]. Further investigations have indicated that the PEMT locus of this transcript is related to the progression of multiple cardiovascular diseases [14]. rs936108-C in the PEMT locus is associated with waist circumference and waist-to-hip ratio, which are surrogate indicators of central obesity [94]. Additionally, RAI1-PEMT-RASD1 is related to coronary artery diseases (CAD)[95] (Table 1); this locus is a common risk factor for ischemic stroke, ischemic large artery stroke, and CAD. rs12936587 on chromosome 17 has been identified as a possible male NREM locus [96], overlapping with the RAI1 gene and containing PEMT1, SREBF1, and RASD1 genes. Most importantly, studies have found a temporal relationship between PEMT gene expression in the human heart and clinical characteristics of heart transplantation (HTx) [97] (Table 1). After the first 3 years of HTx, the level of PEMT mRNA is higher. This discovery laid the foundation for subsequent exploration of the relationship between PEMT and cardiovascular diseases. Elevated plasma VLDLs are an important risk factor for cardiovascular diseases [98], and PC is the predominant phospholipids (PL) component of VLDL. The accumulation of intramyocardial lipids can lead to lipotoxic cardiomyopathy [99]. Using ApoE^-/-^ background mice, Cole et al. [100] reported that PEMT deficiency can reduce TG and cholesterol levels in the plasma and heart, decreasing the risk of atherosclerosis and lipotoxic cardiomyopathy [100] (Table 1). Other groups [101, 102] arrived at similar conclusions using different models. LDL receptors (Ldlr), present on the cell membranes of hepatocytes, can recognize Apo E or ApoB100 lipoprotein, resulting in endocytosis of LDL particles. Ldlr^-/-^ mouse is a well-established atherosclerosis model [103]. The elimination of PC biosynthesis via PEMT knockout significantly attenuates atherosclerosis in Ldlr^-/-^ mice induced by a HFD [100].

S-adenosylhomocysteine (AdoHcy) is another product of the PEMT pathway, in addition to PC. AdoHcy can be hydrolyzed to adenosine and homocysteine (Hcy) [104]. Hyperhomocysteinemia is an independent risk factor for the development of cardiovascular diseases [105, 106]. Deletion of PEMT can decrease circulating Hcy by approximately 50% [107, 108], and re-expression of PEMT in McArdle cells can initiate Hcy secretion into the medium (Table 1). Although there is no direct evidence that PEMT can attenuate cardiovascular diseases by decreasing Hcy, current data indicate the possibility. The plausible factors for PEMT acting on cardiomyocytes may also include the following. (1) Drp1: PC produced by PEMT is the most abundant phospholipid in mitochondria and an important acyl chain donor for the synthesis of cardiolipin. Cardiolipin is structurally dependent on many protein translocases including TOM and plays a fundamental role in cardiovascular health. The dimerization and induction of GTPase-active dyneins (such as Drp1) also require cardiolipin (CL) binding. Oligomerization of Drp1 into a helical structure shrinks the outer membrane, which is necessary to separate damaged mitochondria. The combination of CL and Drp1 enhances its oligomerization and GTP hydrolytic activity, which in turn increases the level of mitochondrial division [109]. Drp1-mediated excessive mitochondrial division contributes to cardiac hypertrophy pathogenesis via reactive oxygen species (ROS) production. Cardiac hypertrophy contributes substantially to overall heart failure. (2) ER stress: The inhibition of PEMT activity is improved in a type 1 diabetes model, characterized by the correction of ER stress associated with diabetic nephropathy [39]. Therefore, based on previous reports, we can hypothesize that PEMT may be upregulated in myocardial injury, such as myocardial hypertrophy, changing the PC/PE ratio and initiating ER stress, whereas low PEMT levels can alleviate ER stress [110]. However, some studies have reported that knocking down PEMT levels can also induce ER stress. Therefore, the role of PEMT in the cardiovascular system requires further investigation. Moreover, the alleviation of myocardial cell injury by knocking down PEMT may be related to the changes in its expression level during myocardial injury. (3) Glucagon: PEMT^-/-^ mice fed with a HFD are insulin resistant (IR), but PEMT^-/-^ mice on high-choline diet may suffer from increased plasma glucagon, along with increased glucagon receptor levels, phosphorylated AMP-activated protein kinase (AMPK), and phosphorylated insulin receptor substrate 1 at serine 307 (IRS1-s307). Pressure overload-induced cardiac hypertrophy and heart failure are associated with generalized insulin resistance and hyperinsulinemia. IRS1 is activated during human heart failure.

### PEMT and aging

Aging affects the physiological functions of living organisms and is mediated by various metabolic pathways [111, 112]. Evidence indicates that dysregulated lipid metabolism contributes to aging via various mechanisms [113-115]. Zhou et al. [116] demonstrated that the absence of PEMT increases the divergence of the metabolome during the aging process of the gastrointestinal tract and liver. The vulnerability of the brain increases rapidly with age, and the lipid fatty acid composition of the brain membrane changes with age [117]. Many studies have found that the production of AdoHcy increases with age, contributing to cerebrovascular and neurodegenerative changes in Alzheimer’s disease [118, 119] (Table 1). Increased AdoHcy inhibits hepatic PEMT activity, leading to a decreased flux of docosahexaenoic acid from the liver to the brain[119]. Indirect evidence has shown that dietary supplementation with PE and PC contributes to lifespan extension in C. elegans [120, 121]. Unsaturated PCs play a protective role in neurodegenerative diseases in humans [121].

### PEMT and others

Several studies have indicated that PEMT plays a vital role in nervous system diseases [122-124], and choline can alter mitosis and neuronal protein expression during fetal brain development [125]. Zhu et al. [122, 126] reported that PEMT deficiency increases progenitor cell mitosis and protein methylation and decreases calretinin levels in the embryonic mouse hippocampus (Table 1). Another study [123] found that these changes are associated with changes in docosahexaenoic acid (DHA)-containing phospholipids in membranes in PEMT^-/-^ mice. Maternal dietary DHA can influence fetal hippocampal maturation. Mills et al. [127] designed a study to determine the relationship between neural tube defect (NTD), choline status, and PEMT polymorphisms. They found that maternal choline concentrations are not strongly associated with NTD risk, while one single nucleotide polymorphism (SNP), PEMT rs7946, is closely linked with NTD. The levels of PC and PE in the brain of patients with Alzheimer’s disease (AD) are significantly decreased, and decreased PEMT activity is accompanied by low compensating ability in brain tissues from AD patients [118].

The PEMT gene is not a conventional tumor-suppressor gene; however, several studies have indicated that an increased expression of PEMT is associated with the suppression of hepatocyte growth [63, 128, 129]. This suggests that PEMT may play a role in the regulation of hepatocellular division as a specific “tumor suppressor.” In hepatocellular carcinoma induced by AFB1, the expression of PEMT2 decreases, and overexpression of PEMT2 partially reverses the growth of hepatoma cells [130] (Table 1). P53, a canonical tumor suppressor, is inactivated by point mutations and accumulates in tumor cells; in contrast to P53, PEMT2 is inactivated by decreased mRNA and protein levels [131]. The activity of PEMT2 may increase after partial hepatectomy[132], indicating that PEMT2 expression can be regulated during proliferation. PEMT is also involved in other cancers, in addition to hepatocellular carcinoma, and current studies have found several important SNPs in both bladder and breast cancers [133, 134].

## Conclusion

PEMT is an important enzyme involved in phospholipid metabolism. Current evidence has provided a clear understanding of its location and structure, its role in catalyzing PE methylation to PC, and the regulation of phospholipid methylation. Many studies have revealed a relationship between PEMT and various pathological conditions. However, several issues need to be addressed. First, with an understanding of the mechanisms of PEMT in pathological conditions, it is possible that pharma-ceutical inhibition of PEMT might protect humans from various diseases. Second, it is necessary to elucidate the regulatory elements of the PEMT gene and identify its transcription factors. Third, it is still unclear whether PC from the PEMT pathway is an important source of hepatic TG and determining this would provide a better understanding of lipid metabolism. Overall, we believe that the investigation of the molecular mechanisms of PEMT will allow a more precise understanding of the pathogenesis of various disorders, providing novel underlying pharmacological targets.
